# Efficient Closed-loop Maximization of Carbon Nanotube Growth Rate using Bayesian Optimization

**DOI:** 10.1038/s41598-020-64397-3

**Published:** 2020-06-03

**Authors:** Jorge Chang, Pavel Nikolaev, Jennifer Carpena-Núñez, Rahul Rao, Kevin Decker, Ahmad E. Islam, Jiseob Kim, Mark A. Pitt, Jay I. Myung, Benji Maruyama

**Affiliations:** 1 Department of Psychology, The Ohio State University, Columbus, OH 43210 USA; 2 UES, Inc., Dayton, OH 45432 USA; 3 Materials and Manufacturing Directorate, Air Force Research Laboratory, Wright-Patterson Air Force Base, Dayton, OH 45433 USA; 4 School of Computer Science and Engineering, Seoul National University, Seoul, 151-742 Korea; 5 Present Address: Cornerstone Research Group, Miamisburg, OH 45342 USA

**Keywords:** Carbon nanotubes and fullerenes, Characterization and analytical techniques, Applied mathematics, Synthesis and processing

## Abstract

A major technological challenge in materials research is the large and complex parameter space, which hinders experimental throughput and ultimately slows down development and implementation. In single-walled carbon nanotube (CNT) synthesis, for instance, the poor yield obtained from conventional catalysts is a result of limited understanding of input-to-output correlations. Autonomous closed-loop experimentation combined with advances in machine learning (ML) is uniquely suited for high-throughput research. Among the ML algorithms available, Bayesian optimization (BO) is especially apt for exploration and optimization within such high-dimensional and complex parameter space. BO is an adaptive sequential design algorithm for finding the global optimum of a black-box objective function with the fewest possible measurements. Here, we demonstrate a promising application of BO in CNT synthesis as an efficient and robust algorithm which can (1) improve the growth rate of CNT in the BO-planner experiments over the seed experiments up to a factor 8; (2) rapidly improve its predictive power (or learning); (3) Consistently achieve good performance regardless of the number or origin of seed experiments; (4) exploit a high-dimensional, complex parameter space, and (5) achieve the former 4 tasks in just over 100 hundred experiments (~8 experimental hours) – a factor of 5× faster than our previously reported results.

## Introduction

On average, it takes 20–30 years to bring a new material from conception to implementation^1,2^. This maturation time is currently hindered by the slow rate of experimentation. In order to meet future technology needs, the speed of materials research must be greatly increased. This problem has been recognized in initiatives such as The Science and Technology 2030 Initiative^3^, Experimentation Campaigns by the National Academies^4^, Accelerated Insertion of Metals^5^, Integrated Computational Materials Science & Engineering^6^ and the Materials Genome Initiative^7^. These initiatives have highlighted the need for accelerating materials development and have proposed that experimentation be replaced with modeling and simulation to achieve research goals. To address this challenge, we have developed the Autonomous Research System (ARES)^8–14^, which uses machine learning (ML) in combination with *in-situ* characterization in a closed-loop fashion to expedite materials synthesis and processing. We have previously demonstrated the ability of ARES towards improving the synthesis of carbon nanotubes (CNTs), which have been at the forefront of nanotechnology for the past two decades^8,10^. Their unique structures – high aspect ratios and diameters around 1 nm – make them attractive for a number of applications such as transistors, sensors, electrical cables, interconnects on microchips, field emitters, thermal interface materials, and quantum computers^15^. However, despite two decades of research and discovery of mechanistic insights^16–23^, large-scale synthesis of CNTs with controlled structures and properties has yet to be achieved^15,24,25^.

Our previous effort involved using a random forest^26^ planner with growth conditions exercised through a genetic algorithm^10^. The planner successfully learned to grow CNTs at targeted growth rates^10^. In the present study, we used Bayesian optimization (BO)^27^ as a planner to maximize CNT growth rates. BO is an optimization algorithm popular in machine learning for finding the global optimum of black-box functions. This type of optimization problem is ubiquitous in many real-world problems such as product design (optimizing the design of an electronic device with the minimum number of trial-and-error processes) and marketing research (optimizing consumer preferences), among others. The BO algorithm starts with an initial “guess” (prior) about a range of possible forms of the underlying unknown function and then sequentially and adaptively refines the guess as new data are observed. The data comprise function values evaluated at a set of selected points, the locations of which are carefully determined by the BO algorithm so as to identify the global optimum with the fewest possible number of function evaluations. BO is overall considered to be the state-of-the-art approach for optimizing unknown functions that are expensive to evaluate, and has been used in many domains, including tuning computational models^28,29^, cognitive science^30^, and computer experiments^31^. In material sciences, BO has been used to optimize compositional design and conduct large-parameter searches^32–34^, synthesize polymers and generate molecular conformers^35,36^, evaluate chemical reactions^37^ and parameterize forcefields^38^.

Here, we discuss the results of two campaigns that use a BO-based planner for ARES to find parameter settings that produced high CNT growth rates. Our results show that BO successfully found regions of high growth rate in the parameter space in a consistent manner in about a hundred experiments (~8 hours of testing). To our knowledge, this work represents the first attempt at using BO for CNT synthesis.

## Methods

### Experimental setup

**Figure 1:**
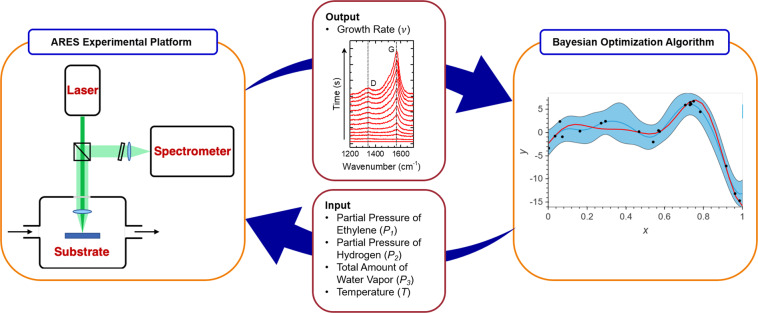
An illustration of the closed-loop experimental scheme. On the left is a schematic of the ARES experimental setup, which includes a 532 nm continuous-wave laser, coupled to a high vacuum chamber and a Raman spectrometer. The CNT growth rates are extracted from the collected data (*in-situ* Raman spectra) and fed into the BO algorithm shown on the right. The algorithm then generates a new set of conditions – the values of the four input variables (${P}_{1},{P}_{2},{P}_{3},T$). These experimental conditions are run by ARES, which records the output data and sends it to the BO planner to update the existing dataset and plan a new experiment.

ARES combines a high-vacuum cold-wall chemical vapor deposition (CVD) chamber, a laser, a Raman spectrometer, and a custom sample substrates containing hundreds of silicon micro-pillars (fabricated by reactive ion etching), as depicted schematically in the upper left of Fig. 1 ^8–14^. A 532 nm laser (between 1–2 W output) is focused at the surface of a catalyst-coated micro-pillar (~10 *μ*m in diameter and height), which heats the pillar instantaneously up to very high temperatures (800–1000 °C) and at the same time serves as the Raman excitation source. For these experiments, we used a 2 nm-thick Co film as the catalyst, deposited (by ion beam sputtering) on to a barrier layer of 10 nm alumina (deposited by atomic layer deposition). The temperature of each pillar is measured from the red-shifted frequency of the Raman peak from the Si micro-pillar. CNT synthesis then takes place by CVD when the gases of interest (*i*.*e*., ethylene, hydrogen, water vapor) are introduced and locally heated in the vicinity of the pillar. Raman spectra are collected continuously during this process, every ~5 seconds for a duration of ~5 minutes, enabling us to monitor the increase in intensity of the CNT Raman peaks (graphitic G band, disorder-induced D band and diameter-dependent low frequency radial breathing modes or RBMs) over time. The integrated intensity of the G band over time provides us an S-shaped growth curve, which is fitted by a Sigmoidal equation and enables us to calculate the maximum growth rate ($\nu$) from the linear portion of the S-shaped fit. After completion of a growth experiment, the data collected is sent to the BO algorithm, which then analyzes and uses the newly incorporated data to plan a new experiment^10^. The process is continuously repeated until the experimental campaign is completed.

Prior to conducting the two BO campaigns, we first generated a seed of experiments which were manually run, consisting of a series of input and output variables as depicted in Fig. 1. Each seed experiment was conducted by varying a set of parameters, including (1) the total system pressure (${P}_{0}$, in Torr), (2) the flow rate of ethylene (${f}_{{C}_{2}{H}_{4}}$, in sccm), (3) the flow rate of hydrogen (${f}_{{H}_{2}}$, in sccm), (4) the total amount of water vapor (${P}_{3}$, in ppm), and (5) the growth temperature ($T$, in °C). The first three parameters were then used to calculate more meaningful variables for the BO algorithm, e.g., (6) the partial pressures of ethylene (${P}_{1}$ in Torr) and (7) the partial pressures of hydrogen(${P}_{2}$ in Torr). In addition to these variables, a final and critical output variable, (8) the maximum growth rate ($\nu$), was recorded from the experimental data and added to the set of variables provided to the BO algorithm. BO then used the quantities extracted from the seed experiments to propose a new set of conditions to run (${P}_{1}$, ${P}_{2}$, ${P}_{3}$, and $T$) and predict the experimental outcome (${\nu }_{pred.}$). The boundaries for the designed parameters were determined experimentally from the physical constraints of the system (*i*.*e*., the range of each mass flow controller). For instance, the total system pressure ranged between 1 Torr to ~750 Torr. Meanwhile, the three gaseous species were limited by the range of each individual mass flow controllers (10–680 sccm for ethylene, 2–100 sccm for hydrogen, and 0.113–2.265 sccm, or 5–100 mg/hr for water).

### Bayesian optimization

Bayesian optimization (BO) is an adaptive sequential design approach for globally optimizing a black-box objective function that is expensive to evaluate^27,39,40^. For our experiments, the objective function to be maximized is the square root of the growth rate ($\sqrt{\nu }$) given the partial pressure of ethylene (${P}_{1}$), partial pressure of hydrogen (${P}_{2}$), total amount of water vapor (${P}_{3}$), and temperature ($T$). BO works by using a pair of ingredients, a *surrogate model* to approximate the objective function to be maximized and an *acquisition function* to quantify the utility of candidate points for evaluation. Put another way, BO uses the surrogate model to explore and make educated guesses about the location of the maximum of the objective function, with the search being guided by the acquisition function. Importantly, the surrogate model is being updated as new observations are made and so is the acquisition function. In the following section, we provide a brief introduction to BO. For a more in-depth review of BO, readers are directed to the work published by Shahriari *et al*.^27^.

#### Gaussian processes

Gaussian processes (GPs) are the most popular choice of surrogate model for BO systems. GPs are a nonparametric, thus model-free, Bayesian modeling approach commonly used for regression and classification problems in the machine learning literature^41^. Being non-parametric, GPs do not assume a defined functional shape found in parametric models. This property endows GPs with a high, virtually unlimited, degree of flexibility that allows them to fit a wide range of data patterns. Historically, GPs were first introduced in the field of geostatistics as a regression technique^42^. Recently, there has been a surge of interest in GPs inspired by the development of powerful numerical approximation methods, such as Markov Chain Monte Carlo^43^ and variational inference^44^.

Formally, a GP is a stochastic (random) process where any subset of random variables forms a Gaussian distribution. Let us use $x$ to denote a data point or vector as the input and $f(x)$ as the output to denote the function that we wish to learn. For a set of observed value pairs $(X,F)$ and a set of unobserved pairs $(\tilde{X},\tilde{F})$, the joint posterior distribution under GP is given by:

1 $$[\begin{array}{c}F\\ \tilde{F}\end{array}]\sim {\mathscr{N}}([\begin{array}{c}\mu \\ \tilde{\mu }\end{array}],[\begin{array}{cc}K(X,X) & K(X,\tilde{X})\\ K(\tilde{X},X) & K(\tilde{X},\tilde{X})\end{array}])$$

where *K* is a kernel function that defines the covariance (*i*.*e*., degree of dependency or similarity) between two function values. Theoretically, any non-negative function can be used as a kernel function and this choice will determine the properties (e.g., smoothness) of the resulting function $f(x)$. Intuitively, a GP can be thought of as an infinite-dimensional Gaussian distribution with the kernel function defining the covariance matrix of this distribution.

The kernel function depends on the distance between two points in the input space. As a result, points that are close together will usually have a higher correlation than those that are far apart. We can use this idea to infer the expected outcome of any point which will produce a smooth function. In the present study, we used the Matern kernel (Rasmussen *et al*.^41^, p. 84), which is defined as:

2 $$K(x,\tilde{X})={\beta }^{2}\left(\,-\frac{{2}^{1-\alpha }}{\Gamma (\alpha )}\right){\left(\frac{\sqrt{2\alpha }(x-\tilde{X})}{l}\right)}^{\alpha }{K}_{\alpha }\left(\frac{\sqrt{2\alpha }(x-\tilde{X})}{l}\right)$$

where *β*, $l$ and *α* are non-negative parameters, $\varGamma$ is the gamma function, and ${K}_{\alpha }$ is the modified Bessel function of the second kind^45^. This kernel function is a popular choice since it is a general-purpose kernel with a high degree of flexibility. For our study, we use the kernel function with $\alpha =5/2,$ also known as Matern52 kernel. This choice is made after trying several different values of $\alpha$ (see section 2.2.3 for detail).

Once these components are defined, we can do regression using the posterior in Eq. (1) to model $\tilde{F}$ using the conditional of the multivariate normal distribution. During inference, we use the maximum a posteriori (MAP) estimation to optimize the kernel parameters $\theta =(\beta ,l)$.

**Algorithm 1:**
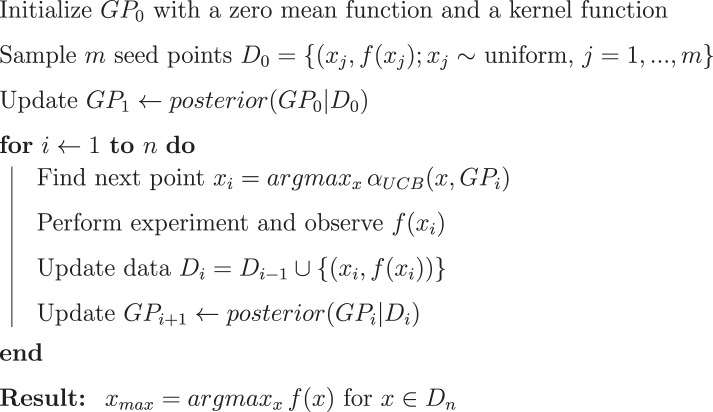
Bayesian Optimization Algorithm.

#### Closed-loop optimization

Although GP regression allows us to model the data available (e.g., predicting growth rate from input parameters), this use of GP does not constitute an optimization algorithm *per se*. Rather, BO makes use of GP models in a different way to extract the necessary information to identify promising points to evaluate as being optimal. This is done by optimizing an acquisition function which takes in information from the posterior GP into a function that is simpler to optimize. The acquisition function then determines the degree of exploration (tendency to probe highly uncertain points in the design space) and exploitation (tendency to try to improve the current best estimate point in the design space).

Many acquisition functions have been proposed with varying degrees of effectiveness depending on the application. A popular acquisition function is the upper confidence bound (UCB) which is defined for a dataset $D={\{({x}_{i},f({x}_{i}))\}}_{i=1}^{n}$ and GP parameters *θ* as:

3 $${\alpha }_{UCB}(x,GP)=\mu (x;D,\theta )+\kappa \cdot \sigma (x;D,\theta )$$

where $\kappa (\, > \,0)$ is a trade-off parameter (also referred to as *jitter*^46^) that controls the balance between exploration and exploitation. Higher values of the $\kappa$ parameter promote more exploration relative to exploitation, and as a result, the search is less likely to get stuck in local optima but tends to converge at a slower pace. The $\mu$ and $\sigma$ parameters in the above equation represent the posterior GP estimate of the mean and standard deviation at a given point $x$. In our experiments and simulations, we adapted the UCB acquisition function for a minimization problem.

**Figure 2:**
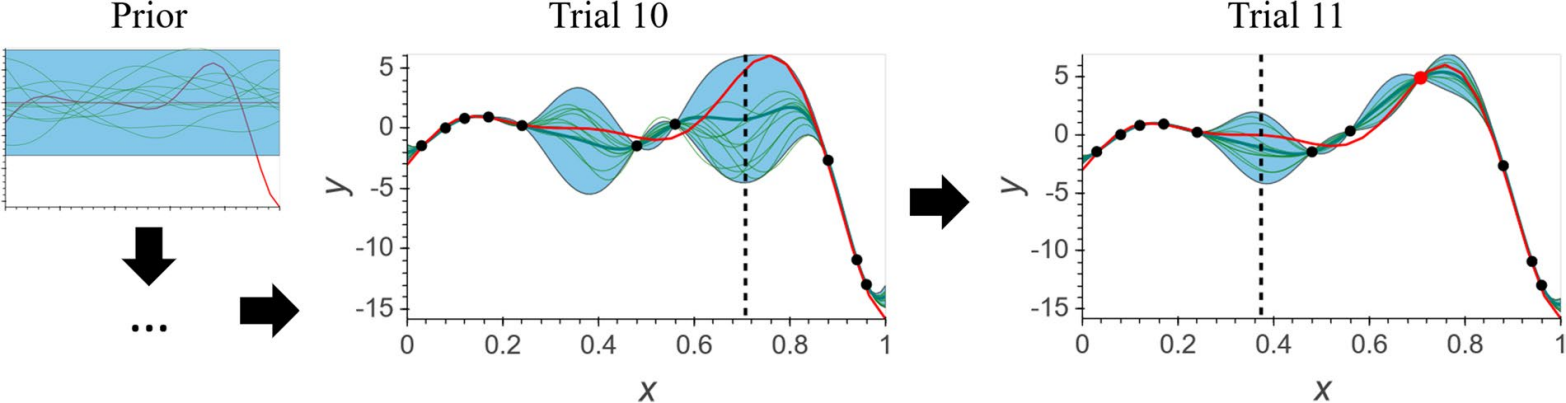
An illustration of Bayesian optimization applied to a toy problem using the Matern52 kernel and the upper confidence bound acquisition function. In each plot, a GP is represented by its mean function (thick green line) and standard deviation (blue area), along with ten random functions sampled from this GP (thin green lines). BO begins with an initial rough approximation (*i*.*e*., prior) about the underlying unknown function and then refines the approximation trial by trial as new observations are made. Observations (black dots) are collected from the ground truth function (red line). At any given trial, BO selects the point with highest acquisition function (dashed line) to evaluate, a new observation (red dot) is made at the next trial, and this sequential adaptive process then repeats itself.

A pseudocode of the BO algorithm is provided in Algorithm 1, and a general scheme of how the algorithm works is illustrated in Fig. 2.

#### Simulations of CNT synthesis

In order to calibrate the BO algorithm and choose among different kernel and acquisition functions, we first ran a set of simulations using data collected in our previous study^10^. We fitted this data set to a generalized additive model implemented in R using tensor product smoothers of every possible pair of variables as its components^47,48^. The resulting model was then used as the ground truth model in our simulations. Within the BO algorithm, the square root of the growth rate ($\sqrt{\nu }$) was used as the output variable (i.e., objective function) to be maximized over the four input variables, (${P}_{1},{P}_{2},{P}_{3},T$) defined earlier. We found, through pilot tests, that the square-root growth rate is a more manageable scale in our implementation than the raw growth rate ($\nu$). Note that the same optimal solution is obtained using either output variable. In addition, random noise was added to the output with a signal-to-noise ratio of 3.

**Figure 3:**
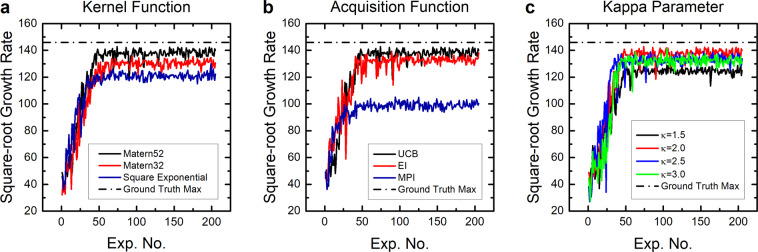
Simulation experiment results. (**a**) Average square-root growth rate as a function of the experiment number for three different choices of the kernel function (Matern52, Matern32 and square exponential), but with the same upper confidence bound (UCB) based acquisition function. (**b**) Results for three different choices of the acquisition function (UCB, expected improvement (EI), and maximum probility of improvement (MPI)), but with the same Matern52 kernel. (**c**) Results for four different values of the parameter $\kappa$ of the UCB acquisition function with the Matern52 kernel. The dashed horizontal line in each panel represents the theoretical maximum (146.0) of the ground truth model.

Figure 3 shows the simulation results. Each line represents an average square-root growth rate over ten independent simulation runs. Ideally, the model should reach the theoretical maximum (dashed line) of the ground truth as closest as possible. Shown on the left panel of the figure is a performance comparison among three different kernel functions: Matern52, Matern32 (with $\alpha =3/2$ in Eq. (2)), and square exponential (with $\alpha \to \infty$), all three combined with the upper UCB based acquisition function. Consistent with previous literature^28^, the Metern52 kernel performed the best. The right panel shows results from three different forms of the acquisition function (e.g., Shahriari *et al*.^27^, pp. 160–162), namely, from the UCB, expected improvement (EI), and maximum probability of improvement (MPI), all combined with the Matern52 kernel. The UCB showed the best result. Based on these simulation results, we used the UCB acquisition function with the Matern52 kernel function in the two campaigns of CNT synthesis described in the next section.

In similar fashion, simulations were run to tune the trade-off parameter $\kappa$ for a few different choices of the parameter, i.e., $\kappa =1.5,2.0,2.5,3.0$. The results of these simulations are presented in Fig. 3c. Here, $\kappa =2.0$ performed the best, although the performance difference was smaller compared to previous manipulations. Thus, we set $\kappa$ to 2.0 for all experiments described in the following section.

## Optimization of CNT Growth rate

Two comparable BO-planned experimental campaigns are presented as validation for the utility of BO in maximizing CNT growth rate. Both campaigns consisted of seeded datasets generated using two distinct methods, and a total of 105 and 104 experiments planned by BO, respectively. For clarity, we labeled these two campaigns as BO-$1$ and BO-$2$. Seed and planned experiments in both campaigns contained the four input variables (${P}_{1}$, ${P}_{2}$, ${P}_{3}$, $T$) and the output variable $\nu$ defined in section 2.1, with the distinction that seed experiments were predetermined to initialize the BO planner whereas BO-planned experiments were provided by BO in an attempt to reach ${\nu }_{max}$ (see Fig. 1). As mentioned earlier, the “BO planner” does not directly optimize the growth rate $\nu$, but instead, it operates on a square root transformation (*i*.*e*., $\sqrt{\nu }$). Further, note that the BO planner was calibrated by performing a set of simulation experiments to determine the best combination of parameter and kernel function settings, as described in section 2.2.3.

The two campaigns contained distinct seeds, which aimed to test the robustness of the algorithm. The seed data set in BO-$1$ contained 25 experiments which were manually selected and run using prior knowledge, *i*.*e*., conditions known to produce successful growths. The seed data set in BO-$2$ was randomly sampled from a uniform distribution to produce 48 unbiased growth conditions, where the list of random experiments was executed by ARES in automatic mode. After receiving the seed data set (containing input and output variables), BO suggested a new set of growth conditions to be tested, along with the corresponding predicted growth rate. ARES then executed the experiment and updated the data set (seed + new experiment) autonomously in a closed-loop fashion.

**Figure 4:**
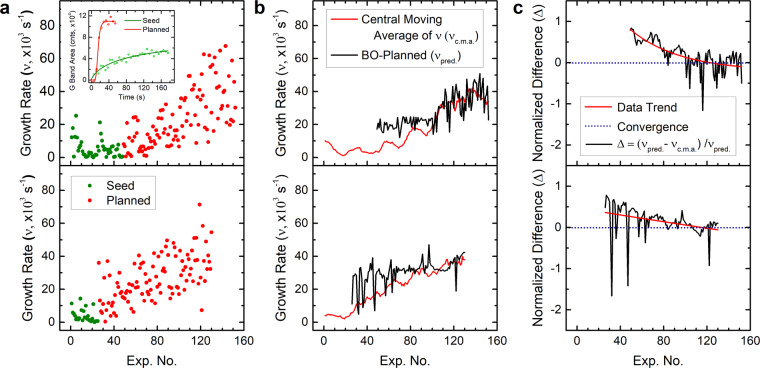
BO increased CNT growth rate ($\nu$) up to a factor of 8 and improved its prediction over time – thus effectively demonstrating learning. (**a**) The raw growth rate of seed and planned experiments for the two BO campaigns, BO-1 (bottom panel) and BO-2 (top panel), increased as BO optimized the objective function $\sqrt{\nu }$. The inset in (**a**) shows example growth curves obtained by ARES from a seed and planned experiment. (**b**) Central moving average of $\nu$ (${\nu }_{c.m.a.}$, calculated using the experimental data in panel (a) with a sample window size of 13 datum points) and predicted growth rates (${\nu }_{pred.}$, provided by BO). (**c**) Normalized difference (Δ) between the central moving average and predicted growth rate for the two campaigns. BO improved the growth rate after only ~105 experiments regardless of how the seed was generated or the number of experiments within the seed.

Figure 4 shows the increase in growth rate over time as the number of experiments increased. The inset in Fig. 4a shows example growth curves obtained by ARES, where the differences in growth rates between the seed and planned growth experiments can be clearly seen. Overall, BO successfully improved the growth rate – up to a factor of 8 – in both experimental campaigns. We emphasize that the seed data used in BO-$1$ was subjectively chosen using human-based prior knowledge, and as such explored a larger parameter space (higher spread noise) than that explored in BO-$2$. Yet, the growth rates in both cases converged within ~100 experiments (confirmed by plateauing of the central moving average) despite the nature of the seed.

It is worth noting that unlike local search algorithms which tend to pick points in close proximity, BO performs global searches on its acquisition function (see Fig. 2). Thus, BO will constantly switch between points with low uncertainties and high expected values (exploitation), and points with high uncertainties (exploration) without a specific path. Figure 4 illustrates this point in the growth rate patterns. Local search algorithms such as hill climbing are expected to produce smooth curves as changes are relatively small between subsequent experiments. In contrast, BO will produce seemingly erratic patterns as it is constantly curious about areas it lacks information on. We argue that this behavior is a key component of the success of BO compared to other approaches. This behavior is regulated by the $\kappa$ parameter defined in Eq. (3). As mentioned earlier, generally speaking, a small $\kappa$ will have a tendency to get stuck in local optima, producing smooth curves, whereas a large $\kappa$ will have a tendency to wander haphazardly, producing rough curves. Thus, it is important to achieve a healthy balance between these two scenarios.

The rapid convergence observed (Fig. 4c) in both BO campaigns (which used two very different seeds) demonstrates the efficiency and robustness of BO and its ability to optimize synthesis conditions within a high-dimensional and complex parameter space. While not completely equivalent, we can contrast the performance of the BO planner to our previously used random-forest planner^10^, which reached the target value only after over 500 experiments. And, while the objective functions were different, we note that BO was able to increase the growth rate up to a factor of 8 and to converge within 5× faster than our previous study. We take this as evidence that BO is able to achieve the goal of optimizing CNT growth rate in a more efficient manner compared to the random forest planner. The rate of learning of BO is also depicted in Fig. 4. In both BO campaigns the experimental growth rates converged to the BO-predicted growth rates after ~100 experimental iterations (Fig. 4c). In other words, the normalized difference Δ between the predicted growth rate and the central moving average (c.m.a.) of the observed growth rate (Δ = $({\nu }_{pred.}-{\nu }_{bluec.m.a.})$/${\nu }_{pred.}$) is gradually reduced to the zero convergence line as BO learned (*i*.*e*., once the planner identifies regions of high growth rate within the parameter space explored and switches to exploitation).

## Discussion

**Figure 5:**
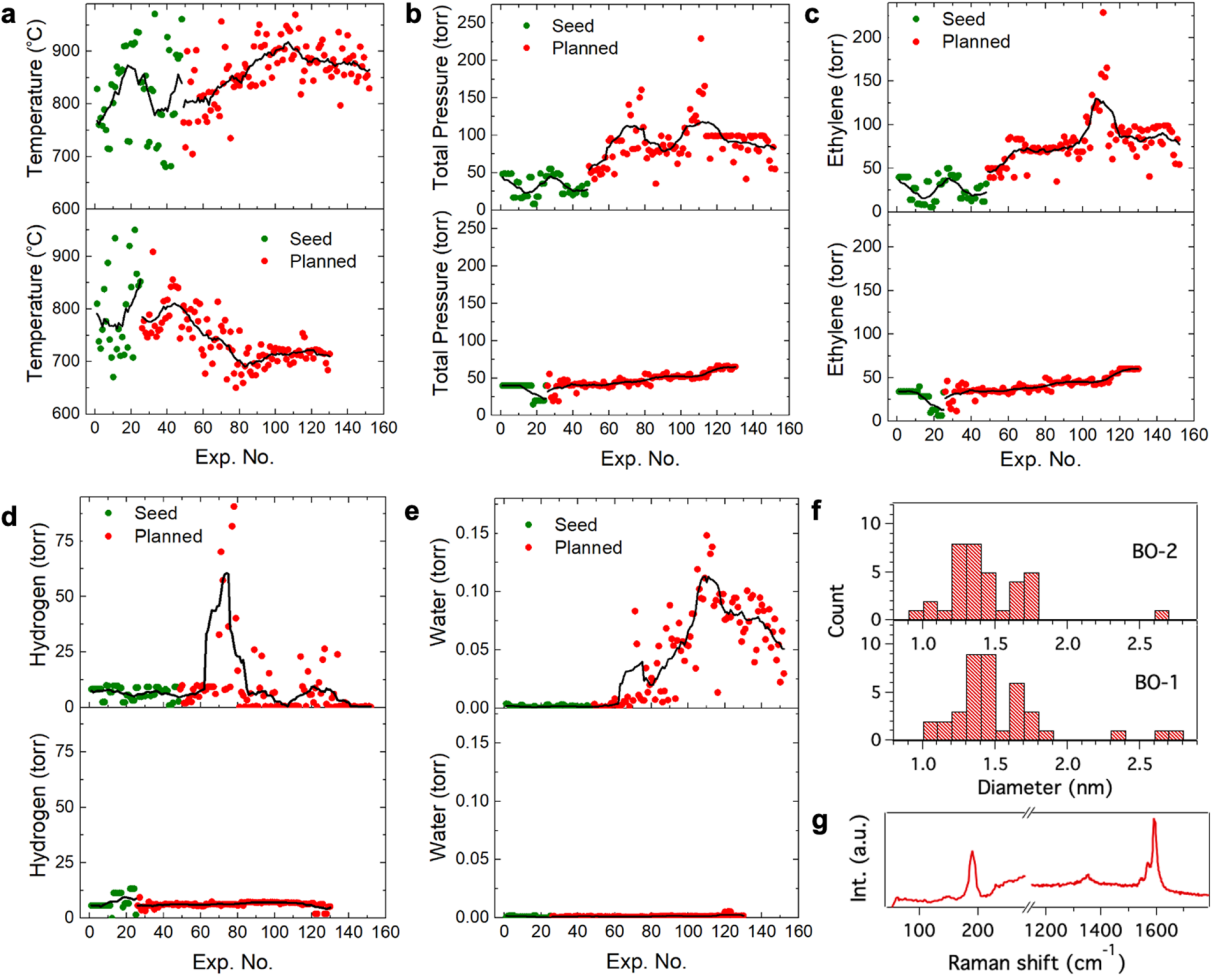
(**a**–**d**) Growth temperature, total pressure, and partial pressures of ethylene, hydrogen and water vapor over the two BO campaigns, BO-1 (bottom panel) and BO-2 (top panel). The seed and planned data points are shown in green and red, respectively. The black traces are the calculated central moving averages (13 datum points), which show the trend in the parameters as the experiments progressed. (**f**) Histogram of CNT diameters calculated from analysis of Raman spectral maps. The data were collected from growth experiments corresponding to high growth rates in BO-1 and BO-2. (**g**) Representative Raman spectrum (excitation at 514.5 nm) showing the low frequency RBM and D and G bands from an individual CNT.

As mentioned above, both BO campaigns produced similar CNT growth rates (Fig. 4) while using different seed data. In order to understand the mechanistic differences between the two growth campaigns, we now take a closer look at the process parameters. Figure 5a–e show the variation in growth temperature, total pressure and partial pressures of ethylene, hydrogen and water vapor for all the experiments in the two BO campaigns (parameters corresponding to BO-1 and BO-2 are in the bottom and top panels of 6, respectively). The green and red data points in Fig. 5 correspond to the seed and planned experiments, respectively, and the black traces are the calculated central moving averages using 13 datum points. Some clear differences can be observed between the parameters for BO-1 and BO-2. The temperature (Fig. 5a) in BO-1 kept decreasing and narrowed to ~700 °C as the experiments progressed, while it increased to ~900 °C in BO-2. The total pressure (Fig. 5b) as well as the pressure of ethylene (Fig. 5c) increased steadily as the experiments progressed, with BO-2 almost double that of BO-1. The hydrogen pressure (Fig. 5d) did not vary significantly across both BO campaigns.

On the other hand, the water level (Fig. 5e) in BO-2 was much higher than in BO-1 (by a few orders of magnitude). The high growth rate experiments in BO-2 were performed with a water vapor pressure range between 0.05 and 0.1 torr (or 600–1000 ppm). These water levels in BO-2 are reminiscent of water-assisted vertically aligned CNT growth (also called super growth)^49^, which is able to produce CNTs at high growth rates^19^. The high water levels in BO-2 are accompanied by higher temperatures and ethylene pressure. Remarkably, the same “super growth-like” growth rates could also be achieved at much lower temperatures (700 vs. 900 °C) by reducing the water pressure to ~1 mTorr (30 ppm). In addition, the ethylene pressure was approximately half the value in BO-2.

We performed further analysis (post-growth) by Raman spectral mapping using two laser excitation wavelengths (514.5 and 633 nm). Raman maps were collected over multiple micropillars that exhibited high growth rates in both BO-1 and BO-2. The low-frequency radial breathing modes (RBMs) are inversely proportional to the nanotube diameter (according to the relation ${\omega }_{RBM}=248/{d}_{t}$, where ${\omega }_{RBM}$ and ${d}_{t}$ are the RBM frequency and diameter, respectively); the nanotube diameters were calculated from the RBM frequencies obtained in the Raman maps, and a histogram of CNT diameters for both BO campaigns is shown in Fig. 5f. For reference, Fig. 5g shows a representative Raman corresponding to a region with the highest growth rate in BO-2. It is clear therein that the samples exhibit a low D-to-G ratio (<0.1) and thus have a low defect density.

The discovery of multiple optima in growth parameters that produce similar growth rates of CNTs with very similar diameter distributions is one of the remarkable outcomes of the BO-run experiments. Further experimental and theoretical work must be done in order to identify more local and global optima that lead to high CNT yields and growth rates. Future efforts will enable BO and other ML algorithms to target more complex growth objectives (*i.e.* diameter and chiral angle selectivity) as well as multiple objectives (*i.e.* defect density and yield).

## Conclusion

In the present study, we introduced Bayesian optimization (BO) as a machine learning algorithm capable of efficient and adaptive optimization of a given objective function within a high-dimensional and complex parameter space in materials science. We demonstrated its use and success in maximizing CNT growth rates. The BO algorithm was able to continuously improve the growth rate up to a factor of 8 over a seed of experiments and to converge after only ~100 experiments, irrespective of how the seed was generated. The efficiency and robustness of BO makes it exceptionally apt for a multitude of tasks in materials research. Future work will enable BO to target multi-objective optimization for high-throughput experimentation and complex, high-dimensional space-exploration. This would allow our system to jointly optimize other values of interest, such as growth yield and purity. Several adaptations of BO have been proposed for this purpose^50,51^, but more work is required to identify the best option.
